# Physical Well-Being of Children and Adolescents during the SARS-CoV-2 Pandemic: Findings from the “Come te la Passi?” Cross Sectional Survey in Bologna, Italy

**DOI:** 10.3390/children9121950

**Published:** 2022-12-12

**Authors:** Aurelia Salussolia, Jacopo Lenzi, Marco Montalti, Flavia Rallo, Martina Paternò, Marta Agosta, Davide Resi, Michela Stillo, Federica Guaraldi, Davide Gori, Laura Dallolio, Alice Masini

**Affiliations:** 1 Department of Biomedical and Neuromotor Sciences, University of Bologna, 40138 Bologna, Italy; 2 Department of Public Health, Bologna Local Health Authority, 40124 Bologna, Italy; 3 Istituto di Ricovero e Cura a Carattere Scientifico delle Scienze Neurologiche di Bologna, 40139 Bologna, Italy

**Keywords:** COVID-19, physical activity, public health, well-being

## Abstract

Background: The COVID-19 had a strong impact on the physical and general well-being of the youngest. In Italy, citizens were forced to change their habits, especially during the national lockdown, causing increased levels of sedentary and unhealthy behaviors. “*Come te la passi?*” was a cross-sectional study aimed at investigating changes in the physical activity levels (PA) and well-being of children and adolescents in the City of Bologna. Methods: An anonymous survey was administered to parents/guardians of children and adolescents aged 6–18 years. Results: 1134 questionnaires were collected during June 2021; 457 (40.3%) were females, and the mean age was 13.0 ± 3.4 years. Regarding the general well-being perception, 61.3% of the participants reported concerns about the future, 46.3% reported sleep difficulties, and 72.8% reported experiencing attention difficulty, with higher percentages among adolescents. Considering the PA frequency, an overall reduction was found, with the percentage of those who rarely did PA and those who frequently did PA both increasing. No gender differences were found. Conclusions: Our findings suggest that the current pandemic has strongly impacted the well-being of children and adolescents. It appears to have primarily affected adolescents, with a significant reduction in PA levels, even after the end of the national lockdown.

## 1. Introduction

To limit the spread of the COVID-19 pandemic during the first months of 2020, most of the countries have closed “non-essential” activities first and, then, working activities, schools, and educational institutions. These measures were estimated to have significantly impacted roughly half of the global student population, not only in terms of education but also on their physical and general well-being [1]. Italy was the second country in the world after China to have experienced the impact of the SARS-CoV-2 outbreak, and, given the rapid spread of the disease, the government imposed school closures simultaneously with the national lockdown from March to September 2020 [2]. Even after the gradual reopening of educational services, many local closures still took place, due to both the local raising of cases and the outbreaks that took place in schools themselves. The implementation of other mandatory virus containment measures, such as stay-at-home orders and social distancing, caused a radical change in the habits and lifestyle of children and adolescents, overall predisposing them to sedentariness and unhealthy behaviors [3,4]. Physical activity (PA) is defined as any bodily movement produced by skeletal muscles that results in energy expenditure [5]. The several benefits of physical activity (PA), including obesity reduction and prevention, bone health, and mental and emotional health in children and adolescents, have now been extensively investigated and demonstrated [6,7]. The recent WHO guidelines recommend that people aged 5–17 perform at least 60 min of moderate-to-vigorous PA (MVPA) per day and vigorous-to-intense PA at least three times per week to maintain good physical and mental health and well-being [8]. In addition, since PA levels during adolescence and adulthood tend to stick to those adopted during early life and childhood [9]. Setting healthy PA behaviors in early life is fundamental to reduce future negative health outcomes such as cardiometabolic disease [10]. Overweight and obesity in children and adolescents are significant concerns in the public health domain, with an increase in prevalence at the European level from 9% to 13% [11]. Emerging data indicate that emergency restrictive measures aimed at controlling the spread of SARS-CoV-2 led to a substantial decline in PA levels in children and adolescents [1]. However, little is known about the mid- and long-term effects of these countermeasures. Based on these premises, the “*Come te la passi?*” (“How is it going?”) survey study aimed at investigating and describing the lifestyle variations concerning PA and general well-being that occurred in children and adolescents after approximately 18 months from the beginning of the pandemic. The hypothesis is to highlight the negative impact of the pandemic on the well-being of both children and adolescents.

## 2. Materials and Methods

### 2.1. Study Design and Participants

A cross-sectional survey study was performed in June 2021 using a convenience sampling approach. An online self-administered questionnaire was sent to the school principals by the Bologna Local Health Authority School Unit—responsible for managing SARS-CoV-2 outbreaks within schools—and then disseminated to parents/guardians of children and adolescents aged <18 years old attending educational services in the Metropolitan City of Bologna (Italy). Study participation was voluntary. Prior to participation, parents/guardians had to sign written informed consent. Parents/guardians were encouraged to answer with the help of and suggestions from children/adolescents. Data were collected anonymously. The study was approved by the School Boards, endorsed by the University of Bologna (Italy), and approved by the University of Bologna Bioethics Committee on 3 June 2021 (Protocol n. 170328). The study was conducted following the Declaration of Helsinki.

### 2.2. Questionnaire and Data Collection

The questionnaire included two distinct sections: the first aimed at collecting participants’ socio-demographic data (i.e., gender, age, attended school, height, and weight); the second aimed at collecting data on children/adolescents well-being and physical activity through 14 questions (closed, multiple, and 5-point Likert scales), using the domains identified by the Pediatric Quality of Life Inventory generic core scales (PEDsQL) [12] and the Physical Activity Questionnaire for Children (PAQ-C) [13]. Specifically, the presence of difficulties/deficits in sleep, attention, and concerns about the future expressed by children/adolescents; the frequency of PA before, during, and after the national lockdown; the frequency of intense PA during the week before answering the questionnaire; the meaning of PA for the child/adolescent; and, finally, potential measures to increase PA were investigated. BMI was calculated using parent’s reported height and weight, and then categorized using Cole cutoff values, taking gender and age into account [14,15].

### 2.3. Statistical Analysis

Numerical variables were reported as mean ± standard deviation (SD), and categorical variables as frequencies and percentages. Descriptive statistics were stratified by age group (6–12 vs. >12 years) and gender. A multilevel mixed-effects generalized ordered probit regression analysis was performed to investigate the change in frequency of child sport participation at each observation time point (during and after the national lockdown) as compared with baseline figures (before the lockdown), with random intercepts for each subject [16]. Generalized probit was preferred over “standard” probit because the parallel regression assumption was rejected by means of the likelihood ratio test [17,18]. Generalized ordered probit is equivalent to a series of binary probit regressions where categories of the dependent variable are cumulatively combined—when there are five categories, as in this study, the first regression contrasts category 1 versus categories 2, 3, 4, and 5; the second regression contrasts categories 1 and 2 vs. 3, 4, and 5; the third regression contrasts categories 1, 2, and 3 vs. 4 and 5; and the fourth regression contrasts categories 1, 2, 3, and 4 vs. 5. Time was treated as a categorical covariate, which resulted in the inclusion of two dummy variables in the model. The outcome was also modeled as a function of time-by-gender and time-by-age group interactions in order to investigate the presence of divergent pre-/post-lockdown increments or decrements between the study groups. Predicted proportions resulting from multilevel modeling were displayed using bar charts and line charts. For categorical data, differences between children and adolescents were also tested using the chi-square test. All analyses were carried out using Stata software, version 15 (StataCorp, 2017; Stata Statistical Software, Release 15: StataCorp LP, College Station, TX, USA). The significance level was set at 0.05, and pairwise deletion of missing data was used.

## 3. Results

### 3.1. Baseline Characteristics

Overall, 1134 questionnaires were collected from 457 (40.3%) female and 677 male (59.7%) participants. Mean age was 13.0 ± 3.4 years. Children (age 6–12 years old) (*n* = 455) represented 40.1% of the study sample and adolescents (age >12 years old) (*n* = 679) represented 59.9%. Overall, the mean BMI was 20.2 ± 3.8 kg/m², lower in children (18.2 ± 3.3 kg/m²) and higher in adolescents (21.5 ± 3.6 kg/m²). 68.4% of the participants (*n* = 772) were of normal weight (children: 63.7%, *n* = 289; adolescents: 71.6%, *n* = 483). With regards to well-being at the time of questionnaire administration, 61.3% (*n* = 695) of the participants reported expressing concerns about the future two or more times per week (66.3%, *n* = 450 among adolescents; 54.8%, *n* = 245 children, *p* < 0.0001), 46.3% (*n* = 525) reported sleep difficulty/deficits two or more times per week (48.9%, *n* = 332 adolescents; 42.4%, *n* = 193 children, *p* = 0.0004), and 72.8% (*n* = 826) reported experiencing attention difficulty/deficits two or more times per week (78.3%, *n* = 532 adolescents; 64.6%, *n* = 294 children, *p* < 0.0001) (Table 1).

Regarding the significance of PA, it was primarily performed to have fun (36.9%, *n* = 418), especially in children (53.6%, *n* = 244), then to work off tension (20.2%, *n* = 229), and to feel good about oneself (18.3%, *n*= 207). The majority of children’s and adolescents’ parents/guardians indicated, among strategies to tackle PA reduction, the creation of additional school time expressly dedicated to PA (53.9%, *n* = 611) and the teaching of simple physical exercises (15.3%, *n* = 174). The nain participants’ characteristics at baseline are reported in (Table 1).

### 3.2. PA Frequency before, during, and after the National Lockdown

Detailed answers to questionnaire items concerning the PA frequency before, during and after the national lockdown and frequency of intense PA, by gender and age group, are shown in (Table 2).

**Figure 1:**
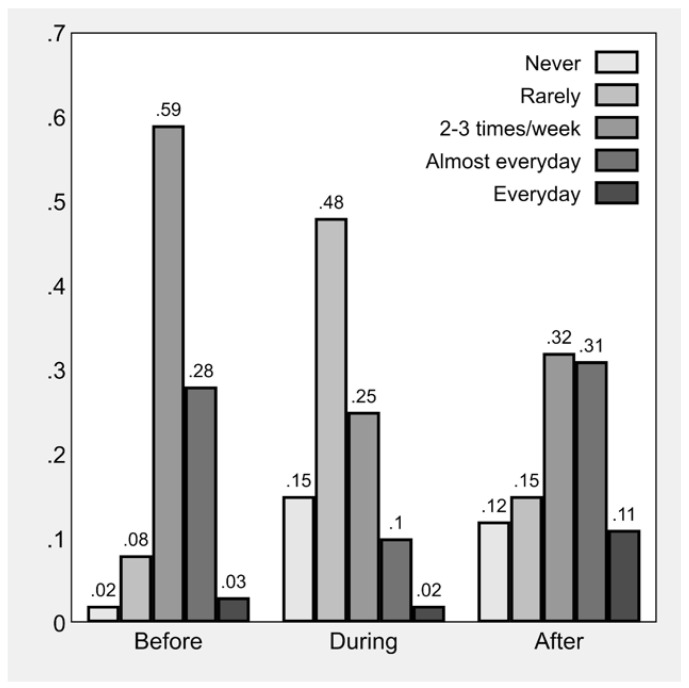
Frequency of child sport participation before, during, and after Italy’s national lockdown (*n* = 1134).

With regards to moderate-to-vigorous PA (MVPA), it emerged that overall 34.7% (*n* = 393) never/rarely performed MVPA the week before the questionnaire administration, with a significantly lower prevalence (22.6%, *n* = 103) in children vs. adolescents (42.7%, *n* = 290). PA frequency before, after, and during the lockdown period and the relative variations are illustrated in (Figure 1). Compared to the previous period, during the national lockdown, PA frequency was reported to be lower. It is worth noting that PA levels after the national lockdown do not match pre-lockdown levels, with a polarization of PA frequency and the percentage of those who never or rarely did PA and those who did PA with a high frequency both increasing, while the percentage of who did PA two or three times per week halved.

**Figure 2:**
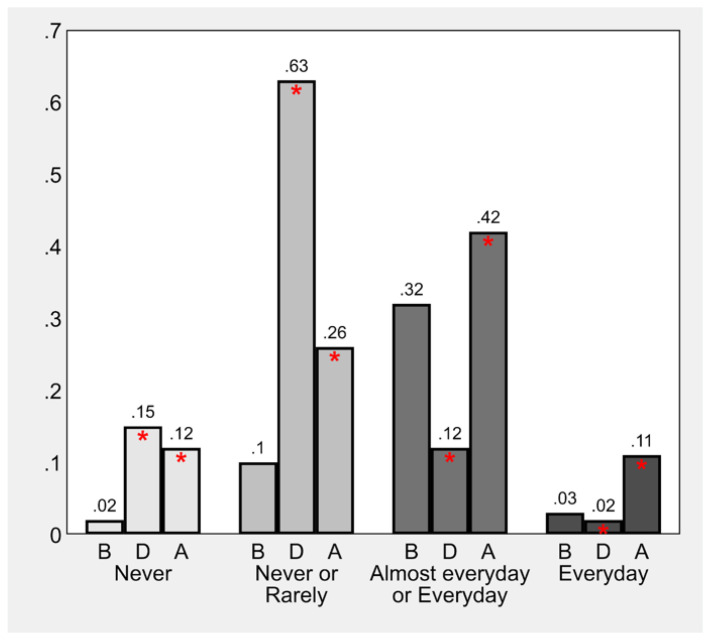
Frequency of child sport participation before (B), during (D), and after (A) Italy’s national lockdown. Point estimates of proportions derive from multilevel mixed-effects generalized ordered probit regression; * indicates a significant increase or decrease relative to B (*p*-value ≤ 0.05).

The analysis of the differences between inactive and physically active children/adolescents is illustrated in (Figure 2). What emerged is that the national lockdown had a massive effect on PA, and that in the subsequent period, PA levels did not recover to previous levels, with a significant difference across all categories (*p*-value ≤ 0.05).

### 3.3. PA Frequency Variation Stratified by Age and Gender

**Figure 3:**
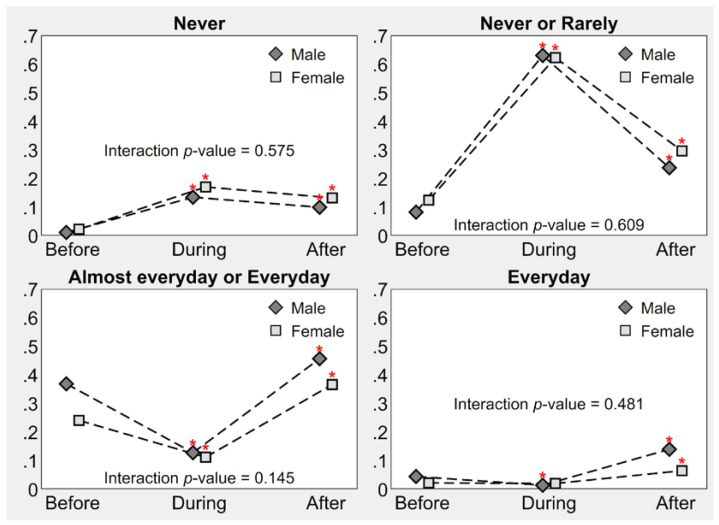
Frequency of child PA before, during, and after Italy’s national lockdown, by gender. Point estimates of proportions derive from multilevel mixed-effects generalized ordered probit regression; * indicates a significant increase or decrease relative to before the national lockdown (*p*-value ≤ 0.05); the interaction *p*-value indicates whether males and females have significantly different increments or decrements in physical activity before and after the national lockdown. *Note*: Six children with missing sex were excluded (*n* = 1128); all estimates were adjusted for age group (≤/>12 years).

Differences in the impact of the national pandemic on PA frequency were also analyzed by stratifying the data by age group and gender. As illustrated in Figure 3, regression analysis showed a significant difference between the proportions of participants’ PA frequency both during and after the lockdown, with the first one significantly decreasing. For the period following the lockdown, the polarization pattern is repeated, with the proportion of both those who rarely do PA and of those who do PA with a high frequency increasing significantly after the national lockdown. These time trends, albeit significant, did not differ based on gender (interaction *p*-values > 0.05).

**Figure 4:**
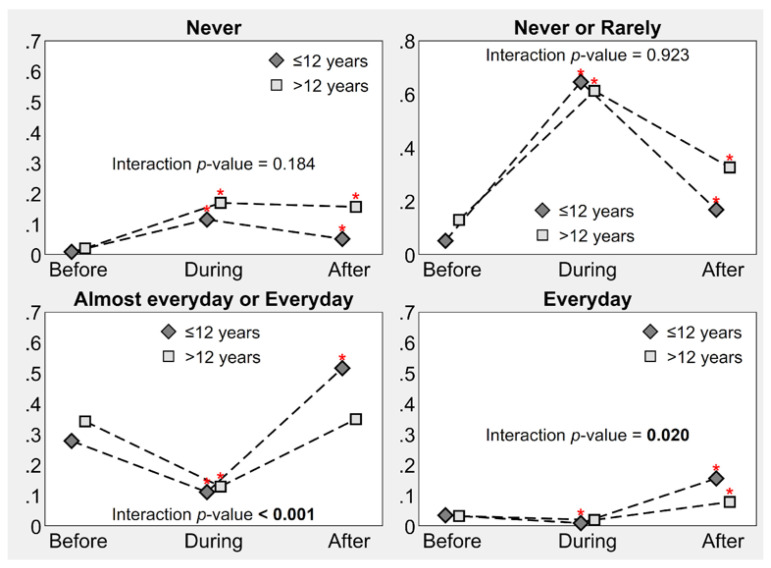
Frequency of child PA before, during, and after Italy’s national lockdown, by age group. Point estimates of proportions derive from multilevel mixed-effects generalized ordered probit regression; * indicates a significant increase or decrease relative to before the national lockdown (*p*-value ≤ 0.05); the interaction *p*-value indicates whether ≤12- and >12-year olds have significantly different increments or decrements in physical activity before and after the national lockdown. Note: All estimates were adjusted for gender.

In addition to the aforementioned analysis, children and adolescents’ PA frequencies were compared, resulting in a difference in high PA frequency categories (almost everyday or everyday: interaction *p*-value < 0.001; everyday: interaction *p*-value = 0.020) (Figure 4). Among children, the increase in PA frequency after the national lockdown was sharper than among adolescents, for whom the percentage remained stable when comparing before and after.

## 4. Discussion

This cross-sectional survey performed in the Metropolitan City of Bologna (Italy) suggested a potential impact of restrictive COVID-19 measures on the well-being and PA levels of youth. In agreement with previous studies, a significant reduction of PA levels during the lockdown [19,20,21] was observed and persisted for a long time after the end of the lockdown itself. Even before the COVID-19 pandemic, both children and adolescents had difficulties following WHO guidelines on PA [8] for both intensity and frequency, and this aspect was further worsened with the lockdown [1,19,20,21,22]. According to the shown data, vigorous PA was performed almost every day or every day only by 44.8% of the children and 32.4% of the adolescents, thus more than half of the participants do not adhere to the WHO recommendations about MVPA. The Italian surveillance system Okkio alla SALUTE [23] reported more positive data; only 20.3% of participants did not reach the PA recommendations. PA modifications seemed to affect adolescents more than children. In particular, adolescents are overall less active than children, and PA reduction is somehow proportional to age [24]. This may be related to the fact that children are more likely to engage themselves in games and activities performed in small spaces at home or in their neighborhoods [25], while adolescents prefer structured team sports, most of which were blocked during the pandemic [26]. As suggested by Steene-Johannessen et al., at least two-thirds of European children and adolescents are insufficiently active and should be of concern for public health authorities. This negative trend before the pandemic was inevitably exacerbated by the measures applied to contain the contagion [27].

A review by Yomoda et al., focusing on data related to the first half of 2020, when lockdown measures were in place in many countries, reported an overall significant decline in PA during the first months of the COVID-19 pandemic in youths, especially in boys and in adolescents [1,28,29]. Differently from other studies reporting the worsening of PA levels, especially in males [21,30], significant differences between males and females were not observed. This difference could be explained by the convenience nature of the sample and by the PA frequency assessment without objective measurements. A study by Maugeri et al. reported a significant reduction in total weekly PA and energy expenditure in children of all ages during the lockdown that negatively affected psychological well-being [31]. For the majority of parents/guardians of both children and adolescents, the main strategies to address the reduction in PA were the implementation of school time dedicated to PA and of physical education, in particular, teaching children simple physical exercises. The importance of promoting physical activity at school, i.e., physically active lessons and active breaks [32,33,34], and in spare time has been increasingly reported. In this line, Steene-Johannessen et al. observed that the onset of age-related lowering of PA seems to occur during the transition between early childhood (preschool) and childhood (primary school), which appears to be a critical period where interventions aimed at preventing a decline in physical activity are important [26]. Moreover, future studies should focus on any changes in the dietary habits of children and adolescents as potential outcomes impacted by COVID-19.

Main study limitations are represented by convenience sampling, data collection based on questionnaires administered to parents/guardians that referred to experiences lived by children/adolescents some months earlier, with a non-negligible risk of recall bias [35], and the absence of objective measures to assess the intensity and type of PA.

## 5. Conclusions

Restrictive measures adopted to counteract the spread of COVID-19 severely impacted youths’ well-being in a relatively short period of time by influencing various aspects of daily life, including the reduction of physical activity, already scarce before the pandemic, with long-lasting effects. Adolescents appeared more damaged than children. Therefore, programs aimed at implementing youth awareness of the importance of a healthy and active lifestyle and the chances of performing physical activity at school and in spare time appear fundamental to preventing further worsening of children’s and adolescents’ well-being and preventing long-term metabolic and cardiovascular diseases. Starting from the needs and suggestions reported by parents/guardians as well as by children and adolescents, several public health measures could be imagined to realize structured programs in the various social contexts, with specific population targets to increase their efficacy.

## Figures and Tables

**Table 1:** Baseline characteristics of the study sample, overall and by age group—Metropolitan City of Bologna (2021). Values are counts (percentages) or mean ± standard deviation.

	All (*n* = 1134)	Age Group	
		6–12 y	>12 y
		(*n* = 455)	(*n* = 679)
Age, y	13.0 ± 3.4	9.6 ± 2.1	15.5 ± 1.5
Female	457 (40.3)	211 (46.4)	246 (36.2)
Weight, kg	53.2 ± 17.9	38.6 ± 12.3	63.1 ± 13.8
Height, m	1.6 ± 0.2	1.4 ± 0.2	1.7 ± 0.1
BMI, kg/m^2^	20.2 ± 3.8	18.2 ± 3.3	21.5 ± 3.6
**BMI, IOTF2**	(*n* = 1128) *	(*n* = 454)	(*n* = 674)
Thinness	96 (8.5)	42 (9.3)	54 (8.0)
Normal weight	771 (68.4)	289 (63.7)	482 (71.5)
Overweight	215 (19.0)	101 (22.2)	114 (16.9)
Obesity	46 (4.1)	22 (4.8)	24 (3.6)
**Concerns about the future**	(*n* = 1134)	(*n* = 455)	(*n* = 679)
Never	204 (18.0)	104 (22.9)	100 (14.7)
Rarely	235 (20.7)	106 (23.3)	129 (19.0)
Two or three times a week	434 (38.3)	175 (38.5)	259 (38.1)
Almost everyday	203 (17.9)	63 (13.8)	140 (20.6)
Everyday	58 (5.1)	7 (1.5)	51 (7.5)
**Sleeping difficulty/deficit**			
Never	336 (29.6)	145 (31.9)	191 (28.1)
Rarely	273 (24.1)	117 (25.7)	156 (23.0)
Two or three times a week	318 (28.0)	138 (30.3)	180 (26.5)
Almost everyday	154 (13.6)	44 (9.7)	110 (16.2)
Everyday	53 (4.7)	11 (2.4)	42 (6.2)
**Attention difficulty/deficit**			
Never	111 (9.8)	58 (12.7)	53 (7.8)
Rarely	197 (17.4)	103 (22.6)	94 (13.8)
Two or three times a week	422 (37.2)	189 (41.5)	233 (34.3)
Almost everyday	309 (27.2)	93 (20.4)	216 (31.8)
Everyday	95 (8.4)	12 (2.6)	83 (12.2)
**PA meaning**			
Having fun	418 (36.9)	244 (53.6)	174 (25.6)
Working off some tension	229 (20.2)	55 (12.1)	174 (25.6)
Feeling good about oneself	207 (18.3)	50 (11.0)	157 (23.1)
Feeling stronger and more energetic	159 (14.0)	52 (11.4)	107 (15.8)
Be more willing to be active	121 (10.7)	54 (11.9)	67 (9.9)
**Interventions to increase PA**			
Establish set times dedicated to PA	611 (53.9)	265 (58.2)	346 (51.0)
Knowing simple exercise	174 (15.3)	74 (16.3)	100 (14.7)
Having online classmates	65 (5.7)	23 (5.1)	42 (6.2)
Being able to exercise with friends	55 (4.9)	24 (5.3)	31 (4.6)
Other	229 (19.3)	69 (15.1)	160 (23.5)

* Six missing data points are due to the participants’ identifying as neither female or male; BMI: body mass index; PA: physical activity.

**Table 2:** Answers to questionnaire items, by gender and by age group (*n* = 1134)—Metropolitan city of Bologna (2021). Values are counts (percentages).

	Gender *		Age Group	
	Female	Male	6–12 y	>12 y
	(*n* = 457)	(*n* = 671)	(*n* = 455)	(*n* = 679)
**Frequency of PA before lockdown**				
Never	11 (2.4)	7 (1.0)	3 (0.7)	16 (2.4)
Rarely	46 (10.1)	45 (6.7)	18 (4.0)	73 (10.8)
Two or three times a week	288 (63.0)	376 (56.0)	313 (68.8)	354 (52.1)
Almost everyday	101 (22.1)	217 (32.3)	109 (24.0)	211 (31.1)
Everyday	11 (2.4)	26 (3.9)	12 (2.6)	25 (3.7)
**Frequency of PA during lockdown**				
Never	77 (16.8)	89 (13.3)	49 (10.8)	119 (17.5)
Rarely	205 (44.9)	336 (50.1)	249 (54.7)	296 (43.6)
Two or three times a week	121 (26.5)	163 (24.3)	113 (24.8)	171 (25.2)
Almost everyday	44 (9.6)	75 (11.2)	41 (9.0)	78 (11.5)
Everyday	10 (2.2)	8 (1.2)	3 (0.7)	15 (2.2)
**Frequency of PA during last week**				
Never	61 (13.3)	69 (10.3)	21 (4.6)	112 (16.5)
Rarely	76 (16.6)	92 (13.7)	53 (11.6)	116 (17.1)
Two or three times a week	153 (33.5)	204 (30.4)	149 (32.7)	209 (30.8)
Almost everyday	134 (29.3)	215 (32.0)	168 (36.9)	182 (26.8)
Everyday	33 (7.2)	91 (13.6)	64 (14.1)	60 (8.8)
**Frequency of MVPA during last week**				
Never	76 (16.6)	86 (12.8)	30 (6.6)	134 (19.7)
Rarely	118 (25.8)	109 (16.2)	73 (16.0)	156 (23.0)
Two or three times a week	122 (26.7)	194 (28.9)	148 (32.5)	169 (24.9)
Almost everyday	112 (24.5)	197 (29.4)	147 (32.3)	162 (23.9)
Everyday	29 (6.3)	85 (12.7)	57 (12.5)	58 (8.5)

* Six missing data points are due to the participants’ identifying as neither female or male; PA: physical activity; MVPA: moderate-to-vigorous physical activity.

## Data Availability

Availability of data and materials: The data presented in this study are available on request from the corresponding author. The data are not publicly available due to ethical and privacy reasons.
